# Risk of Bacteremia in Febrile Children and Young Adults With Sickle Cell Disease in a Multicenter Emergency Department Cohort

**DOI:** 10.1001/jamanetworkopen.2023.18904

**Published:** 2023-06-20

**Authors:** Stephen Rineer, Patrick S. Walsh, Luke R. Smart, Nusrat Harun, David Schnadower, Matthew J. Lipshaw

**Affiliations:** 1 Division of Emergency Medicine, Cincinnati Children’s Hospital Medical Center, Cincinnati, Ohio; 2 Section of Emergency Medicine, Children’s Hospital of Wisconsin, Department of Pediatrics, Medical College of Wisconsin, Milwaukee; 3 Division of Hematology, Cincinnati Children’s Hospital Medical Center, Cincinnati, Ohio; 4 Department of Pediatrics, University of Cincinnati College of Medicine, Cincinnati, Ohio; 5 Division of Biostatistics and Epidemiology, Cincinnati Children’s Hospital Medical Center, Cincinnati, Ohio

## Abstract

**Importance:**

Bacteremia is a major cause of morbidity and mortality in children and young adults with sickle cell disease (SCD), but among those presenting to the emergency department (ED) with fever, the absolute risk of, risk factors associated with, and outcomes of bacteremia are poorly defined.

**Objective:**

To obtain contemporary data on the absolute risk of, risk factors associated with, and outcomes associated with bacteremia in children and young adults with SCD presenting to the ED with fever.

**Design, Setting, and Participants:**

A multicenter retrospective cohort study was conducted of individuals with SCD younger than 22 years (young adults) presenting to EDs within the Pediatric Health Information Systems database from January 1, 2016, to December 31, 2021, with fever (identified by diagnostic codes for fever or the collection of blood samples for cultures and intravenous antibiotic administration). Data analysis was performed from May 17 to December 15, 2022.

**Main Outcomes and Measures:**

The risk of bacteremia (defined by diagnostic coding) was identified in these children and young adults, and univariate analyses and multivariable regression were used to examine patient-level factors and bacteremia.

**Results:**

A total of 35 548 encounters representing 11 181 individual patients from 36 hospitals were evaluated. The median age of the cohort was 6.17 (IQR, 2.36-12.11) years and 52.9% were male. Bacteremia was present in 405 encounters (1.1%, 95% CI, 1.05%-1.26%). A history of bacteremia, osteomyelitis, stroke, central line–associated bloodstream infection (CLABSI), central venous catheter, or apheresis was associated with the diagnosis of bacteremia, while age, sex, hemoglobin SC genotype, and race and ethnicity were not. In the multivariable analysis, individuals with a history of bacteremia (odds ratio [OR], 1.36; 95% CI, 1.01-1.83), CLABSI (OR, 6.39; 95% CI, 3.02-13.52), and apheresis (OR, 1.77; 95% CI, 1.22-2.55) had higher odds of bacteremia.

**Conclusions and Relevance:**

The findings of this large cohort study suggest that bacteremia in children and young adults with SCD presenting with fever is rare. A history of invasive bacterial infection, CLABSI, or a central line appears to be associated with bacteremia, while age and SCD genotype are not.

## Key Points

**Question:**

What are the risks and outcomes associated with the receipt of a diagnostic code for bacteremia in children and young adults with sickle cell disease presenting to the emergency department with fever?

**Findings:**

In this cohort study including 35 548 encounters representing 11 181 individual patients with sickle cell disease, bacteremia was present in 1.1% of the encounters from 36 hospitals. In the multivariable analysis, individuals with a history of bacteremia–associated bloodstream infection and apheresis had higher odds of bacteremia.

**Meaning:**

The results of this study suggest that a new episode of bacteremia is an uncommon diagnosis for children and young adults with sickle cell disease presenting to the emergency department with fever; a new diagnosis of bacteremia was significantly more likely if there was a history of a central line placement or bacteremia.

## Teaser

This cohort study examines the diagnosis of bacteremia in children and youths with sickle cell disease who present to the emergency department with fever.

## Introduction

Sickle cell disease (SCD) is the most common genetic disease worldwide, with an estimated 400 000 children born with it globally each year, 2000 of whom are born in the US.^1^ Bacteremia and invasive bacterial infections are important causes of death in children and young adults with SCD, especially those younger than 5 years, due to the functional asplenia,^2^ immune system defects,^3^ and anatomic predisposition toward osteomyelitis inherent to the underlying disease.^4^ Before development of the conjugate pneumococcal vaccine and hydroxyurea treatment, bacteremia was common, and the likelihood of death from an episode of bacteremia was 15% to 20%.^5^ Given the historical high risk of mortality due to bacteremia, guidelines in the US recommend prompt emergency department (ED) evaluation for fever higher than 38.5 °C and standardized care for this high-risk population, including the collection of laboratory studies, blood cultures, and the administration of antibiotics.^3,6^

The care of children and young adults with SCD has improved substantially during the past 40 years with implementation of penicillin prophylaxis, progressive improvements in vaccine technology, and approval of disease-modifying therapy. Previous studies have reported a decrease in the community incidence of bacteremia after the introduction of penicillin prophylaxis in the 1980s and more recent developments in conjugated pneumococcal and meningococcal vaccines; however, the current rates of and risk factors for bacteremia in those who present to the ED with a fever are unknown.^7,8,9,10,11^ These earlier studies have been small and underpowered to describe precise outcome estimates and associated risk factors. A single meta-analysis of small studies described a bacteremia rate of 1.9% in this population subsequent to the introduction of the 7-valent pneumococcal conjugate vaccine,^12^ but to our knowledge, this has yet to be confirmed in a large nationwide cohort.

The primary objectives of this study were therefore to examine the risk of bacteremia, associated risk factors, use of resources, and clinical outcomes in children and young adults with SCD presenting to the ED with fever in a large multicenter cohort. Describing precise risk estimates and risk factors associated with bacteremia in children and young adults with SCD and fever could inform clinicians and families regarding the need for ED visits, diagnostic workup, use of antibiotics, and hospital admission.

## Methods

### Study Design and Setting

We performed a multicenter retrospective cohort study using the Pediatric Health Information Systems (PHIS) database. As an administrative database, PHIS contains encounter-level data from approximately 50 tertiary care US pediatric hospitals affiliated with the Children’s Hospital Association. The Children’s Hospital Association and participating hospitals ensure data quality and reliability, and data are subjected to a number of reliability and validity checks.^13^ We included the sites that contributed complete clinical and billing data for the entire study period. Data included from the PHIS do not describe discrete biomedical values (eg, vital signs, laboratory test values) but instead rely on demographic characteristics documented in the medical records (eg, medical history), physician-supplied diagnoses (ie, *International Statistical Classification of Diseases and Related Health Problems, Tenth Revision* [*ICD-10*] codes), and billing data for any procedures or tests that were completed during the hospital stay. This study was determined to be exempt from institutional review and the requirement for informed consent at Cincinnati Children’s Hospital Medical Center in accordance with applicable regulations and institutional policy. This study follows the Strengthening the Reporting of Observational Studies in Epidemiology (STROBE) reporting guideline for cohort studies.

### Study Population

We included children and adults younger than 22 years with a diagnosis of SCD (*ICD-10* code D57, excluding sickle cell trait [code D57.3]) presenting to the ED with fever (codes R50.81 and R50.9) or undergoing diagnostic testing and treatment consistent with fever (performance of blood culture and treatment with parenteral antibiotics on the day of ED presentation) between January 1, 2016, and December 31, 2021.^6,14^ We used diagnostic codes and clinical interventions to define cases because vital signs and clinical notes are unavailable in the PHIS data set.

We excluded further encounters for children and young adults that occurred within 3 days of an index encounter, as these repeat visits were part of our primary outcome measure. We excluded encounters resulting in hospital transfer as we were unable to determine clinical outcomes for those encounters. We excluded children and young adults with a history of bone marrow transplant given this group’s unique risk factors for infection.

### Measurements

We extracted patient demographic characteristics, including age, sex, race and ethnicity, hospital, US census region, and year of encounter. Race and ethnicity is included as standard practice with SCD since it has a genetic basis. We extracted all encounter diagnoses for the included encounters and previous encounters in the PHIS database. We specifically evaluated for history of bacteremia, bone marrow transplant, osteomyelitis, splenectomy, stroke, acute chest syndrome (ACS), sepsis,^15^ central line–associated bloodstream infection (CLABSI), central venous catheter (CVC) placement, or treatment with apheresis. These diagnoses were chosen a priori as potential risk factors for bacteremia.^12^ Patients were classified as having a history of a diagnosis if it was present during an encounter within the PHIS database that preceded the index encounter. Both *ICD-9* and *ICD-10* codes used for diagnosis definitions are shown in the eAppendix in Supplement 1. We extracted ED interventions including chest radiograph, computed tomography, magnetic resonance imaging, complete blood cell count, erythrocyte sedimentation rate, C-reactive protein, procalcitonin, reticulocyte count, blood culture, urinalysis, and cerebrospinal fluid assessment. Laboratory results were not assessed as they are not contained within the PHIS database. We extracted information about patient outcomes including discharge from the ED, admission, intensive care unit (ICU) admission, length of stay, use of vasoactive medications, intubation, and ED return visit or hospital readmission within 3 days. Length of stay was calculated as the number of calendar days between admission date and discharge date. Because the PHIS database includes calendar date for charges but not time or location, we included all charges that occurred on hospital day 0 or day 1 to ensure that ED encounters crossing midnight were included, a method that has been used in previous ED-based PHIS studies.^14,16^

### Outcomes

Our primary outcome was the diagnosis of bacteremia (*ICD-10* code R78.81). We included diagnoses both from the index ED encounter and for encounters within 3 days following an ED discharge to account for some individuals potentially being discharged home with blood culture results pending and then returning for a second encounter if the initial culture was positive. Secondary outcomes included resource use at the index visit (radiography, laboratory studies, and hospital admission), and clinical outcomes (concomitant infections or serious diagnoses [specifically, ACS, septic joint, meningitis, osteomyelitis, and urinary tract infection], length of stay, and ICU admission).

### Statistical Analysis

Statistical analysis was conducted from May 17 to December 15, 2022. We described cohort characteristics using frequencies with proportions for categorical data and median and IQR for continuous data. We evaluated differences in clinical characteristics and outcomes between patients with and without bacteremia using a χ^2^ or Fisher exact test as appropriate for categorical variables and a nonparametric Wilcoxon rank sum test for continuous variables; level of statistical significance determined with 2-sided testing was *P* < .05. We constructed a multivariable logistic regression model first including biologically plausible variables and used a backward elimination approach by removing variables with a value of *P* > .20 from the multivariable model. We assessed collinearity by investigating the correlation matrix of the regression coefficients in a multivariable logistic regression model with all risk factors. We constructed another model combining variables that are clinically related (eg, bacteremia, sepsis, and CLABSI; apheresis and CVC). We used the Akaike information criteria^17^ to assess goodness of fit of all fitted models. The final model was chosen to be the one with the smallest Akaike information criteria value. To account for the correlation in estimates arising from repeated visits and hospital clustering, we fit a generalized linear model with a logit link including a separate random effect for each unique patient from each hospital. We conducted a sensitivity analysis excluding children who did not undergo a blood culture at the index visit and compared characteristics of children and young adults with bacteremia admitted and discharged at the index visit. Statistical analyses were conducted using R, version 4.2.1 (R Foundation for Statistical Computing) and SAS, version 9.4 (SAS Institute Inc).

## Results

### Study Population

We identified 98 684 ED encounters for children and young adults with SCD from 36 hospitals over the 5-year study period. After exclusions, we were left with a final analytic cohort of 35 548 encounters (Figure) representing 11 181 individual patients. The median age of the cohort was 6.17 (IQR, 2.36-12.11) years, 52.9% were male, and 47.1% were female.

**Figure.**
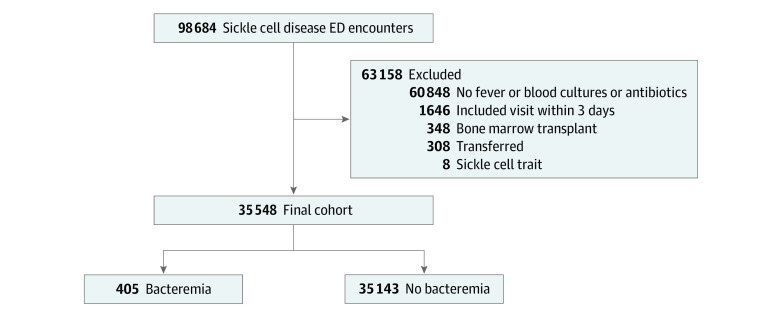
Study Flow Diagram Some children had more than 1 exclusion criterion. ED indicates emergency department.

### Primary Outcome

Bacteremia was diagnosed within 3 days of the index visit in 405 of 35 548 encounters (1.1%; 95% CI, 1.03%-1.26%), representing 371 unique children and young adults. Of the diagnoses of bacteremia, 278 occurred during the index encounter and 127 occurred at return visits within 3 days following discharge from the ED. There were no significant differences between children and young adults with and without bacteremia in terms of age, sex, race and ethnicity, insurance type, and SCD genotype. In univariate analyses, children and young adults with a history of bacteremia, osteomyelitis, stroke, CLABSI, central venous catheter placement, or apheresis were more likely to have a new diagnosis of bacteremia (Table 1). In the multivariable model, a history of bacteremia (odds ratio [OR], 1.36; 95% CI, 1.01-1.83; *P* = .04), CLABSI (OR, 6.39; 95% CI, 3.02-13.52; *P* < .001), and apheresis (OR, 1.77; 95% CI, 1.22-2.55; *P* < .001) was associated with a diagnosis of bacteremia, whereas age, sex, and genotype were not (Table 2). In our sensitivity analysis excluding individuals who did not receive a blood culture at the index visit, results were similar except that a history of osteomyelitis became associated with bacteremia (OR, 2.01; 95% CI, 1.17-3.26; *P* = .01) (eTable 1 in Supplement 1). Individuals with the outcome of bacteremia who were admitted were similar to individuals with bacteremia who were initially discharged from the ED in terms of all demographic factors and aspects of medical history except history of CLABSI (4.4% of admitted individuals vs 0% of discharged individuals; *P* = .01) (eTable 2 in Supplement 1).

**Table 1.** Sickle Cell Disease Cohort Characteristics and Outcomes

Characteristic	No. (%)			*P* value
	Overall	No bacteremia	Bacteremia	
No. of encounters	35 548	35 143	405	NA
Age, y				
Median (IQR)	6.17 (2.35-12.10)	6.17 (2.36-12.10)	6.43 (2.53-13.32)	.42
Range				
<1	3356 (9.4)	3321 (9.4)	35 (8.6)	.39
1-5	11 992 (33.7)	11 861 (33.8)	131 (32.3)	
6-10	8616 (24.2)	8515 (24.2)	101 (24.9)	
11-18	9178 (25.8)	9077 (25.8)	101 (24.9)	
19-21	2406 (6.8)	2369 (6.7)	37 (9.1)	
Sex				
Male	18 816 (52.9)	18 595 (52.9)	221 (54.6)	.54
Female	16 732 (47.1)	16 548 (47.1)	184 (45.4)	
Race				
Black	32 959 (92.7)	32 574 (92.7)	385 (95.1)	.11
White	828 (2.3)	819 (2.3)	9 (2.2)	
Other^a^	1761 (5.0)	1750 (5.0)	11 (2.7)	
Ethnicity				
Not Hispanic or Latino	33 482 (94.2)	33 091 (94.2)	391 (96.5)	.05
Insurance type				
Public	26 477 (74.5)	26 163 (74.4)	314 (77.5)	.15
Private	7929 (22.3)	7845 (22.3)	84 (20.7)	
Other or unknown	1142 (3.2)	1135 (3.2)	7 (1.7)	
Genotype				
HbSS	27 687 (77.9)	27 389 (77.9)	298 (73.6)	.20
HbSC	4950 (13.9)	4884 (13.9)	66 (16.3)	
HbS-thalassemia^b^	2286 (6.4)	2253 (6.4)	33 (8.1)	
Other/multiple	625 (1.8)	617 (1.8)	8 (2.0)	
Concomitant diagnoses				
Acute chest syndrome	8116 (22.8)	8031 (22.9)	85 (21.0)	.37
UTI	492 (1.4)	473 (1.3)	19 (4.7)	<.001
Osteomyelitis	172 (0.5)	166 (0.5)	6 (1.5)	<.001
Septic arthritis	39 (0.1)	36 (0.1)	3 (0.7)	.001
Meningitis	7 (<0.1)	6 (<0.1)	1 (0.2)	.08
Medical history and patient characteristics				
Acute chest syndrome	20 051 (56.4)	19 819 (56.4)	232 (57.3)	.72
Splenectomy	3271 (9.2)	3237 (9.2)	34 (8.4)	.57
Bacteremia	3300 (9.3)	3230 (9.2)	70 (17.3)	<.001
Apheresis	2029 (5.7)	1984 (5.6)	45 (11.1)	<.001
Sepsis	1521 (4.3)	1497 (4.3)	24 (5.9)	.10
Central venous catheter placement	1464 (4.1)	1434 (4.1)	30 (7.4)	<.001
Stroke	898 (2.5)	880 (2.5)	18 (4.4)	.01
Osteomyelitis	656 (1.8)	640 (1.8)	16 (4.0)	<.001
CLABSI	98 (0.3)	86 (0.2)	12 (3.0)	<.001
Outcomes				
3-d Return visit	2816 (8.0)	2668 (7.6)	148 (36.5)	<.001
Admit at 3-d return visit	1473 (4.1)	1371 (3.9)	102 (25.2)	<.001
Length of stay, median (IQR), d	1.0 (1.0-3.0)	1.0 (1.0-3.0)	2.0 (1.0-5.0)	<.001
ICU admission	1080 (3.1)	1058 (3.0)	22 (5.4)	.01
Admission	19 665 (55.3)	19 403 (55.2)	262 (64.7)	<.001
Vasopressor use	184 (0.5)	180 (0.5)	4 (1.0)	.35
Intubation	133 (0.4)	133 (0.4)	0	.41

Abbreviations: CLABSI, central line–associated bloodstream infection; Hb, hemoglobin; ICU, intensive care unit; NA, not applicable; UTI, urinary tract infection.

^a^ Other races include American Indian (12 [<0.1%]), Asian (48 [<0.1%]), multiple (303 [0.9%]), Pacific Islander (11 [<0.1%]), and other (1010 [2.8%]), including 377 unknown.

^b^ HbS-β zero or HbS-β plus.

**Table 2.** Risk Factors for Bacteremia in Multivariable Model^a^

Characteristic	OR (95% CI)	*P* value
Age, y		
<1	1.01 (0.70-1.47)	.94
1-5	0.98 (0.78-1.24)	.89
>5	1 [Reference]	NA
Sex		
Female	1.07 (0.87-1.31)	.53
Male	1 [Reference]	NA
Genotype		
HbSC	0.76 (0.58-1.00)	.05
HbS thalassemia^b^	0.72 (0.50-1.05)	.09
Other/multiple	0.83 (0.41-1.71)	.62
HbSS	1 [Reference]	NA
Previous diagnoses		
Bacteremia	1.36 (1.01-1.83)	.04
Osteomyelitis	1.71 (0.97-3.00)	.06
Splenectomy	0.80 (0.55-1.16)	.23
Sepsis	1.17 (0.75-1.84)	.49
CLABSI	6.39 (3.02-13.52)	<.001
CVC placement	1.25 (0.81-1.92)	.31
Apheresis	1.77 (1.22-2.55)	<.001

Abbreviations: CLABSI, central line–associated bloodstream infection; CVC, central venous catheter; Hb, hemoglobin; NA; not applicable; OR, odds ratio.

^a^ Generalized linear mixed model accounting for repeat encounters and nested within hospitals.

^b^ HbS-β zero or HbS-β plus.

### Secondary Outcomes

The most common concomitant serious diagnoses identified at the same time as fever were ACS (8116 [22.8%]) and urinary tract infection (492 [1.4%]). Meningitis (7 [<0.1%]), osteomyelitis (172 [0.5%]), and septic arthritis (39 [0.1%]) were rare. Bacteremia was present in 19 of 492 individuals (3.9%) with concomitant urinary tract infection and 85 of 8116 individuals (1.0%) with ACS (Table 1). Individuals with bacteremia were more likely to be admitted (64.7% vs 55.2%; *P* < .001) and be admitted to the ICU (5.4% vs 3.0%; *P* = .01) compared with those without bacteremia. Median length of stay was longer in children and young adults with bacteremia (2.0; 95% CI, 1.0-5.0 vs 1.0; 95% CI, 1.0-3.0 days; *P* < .001) (Table 1). While 21 of 278 children and young adults (7.6%) hospitalized and diagnosed with bacteremia during the index visit were admitted to the ICU, only 1 of 127 individuals (0.8%) initially discharged from the ED but diagnosed with bacteremia within 72 hours was admitted to the ICU at revisit.

### Resource Use in the ED

Emergency department resource use for children and young adults with SCD presenting to the ED with fever is reported in Table 3. Testing with complete blood cell count and reticulocyte count was done in more than 95% of the encounters. Chest roentgenography was performed in 68.5% of the encounters, a blood culture was obtained in 87.6%, urine studies were performed in 29.1%, and cerebrospinal fluid was obtained in 0.3%. Computed tomography and magnetic resonance imaging were rarely used in the ED setting. Samples for procalcitonin, C-reactive protein, and erythrocyte sedimentation rate testing were sent in less than 10% of the encounters.

**Table 3.** Emergency Department Resource Use

Characteristic	No. (%)
No.	35 548
Complete blood cell count	34 717 (97.7)
Reticulocyte count	34 036 (95.7)
Blood culture	31 155 (87.6)
Chest roentgenogram	24 344 (68.5)
Urine studies	10 342 (29.1)
C-reactive protein	3142 (8.8)
Procalcitonin	1582 (4.5)
Erythrocyte sedimentation rate	933 (2.6)
Computed tomography	816 (2.3)
Magnetic resonance imaging	460 (1.3)
Cerebrospinal fluid	115 (0.3)

## Discussion

In this large multi-institutional cohort of children and young adults with SCD presenting to the ED with fever during 2016 to 2021, the risk of bacteremia was 1.1%. Individuals with a history of bacteremia, CLABSI, or receiving apheresis had higher odds of a new diagnosis of bacteremia. We found no significant differences in the risk of bacteremia across age or differences in sickle cell genotype. Children and young adults discharged at the index visit and subsequently admitted with a diagnosis of bacteremia within 3 days rarely required ICU-level care.

To our knowledge, this is one of the largest studies to date describing the risk of bacteremia in this population. Our results are similar to small single-center studies from the past 10 years that have reported a wider range of bacteremia (0.3%-4.2%, with a weighted average of 1.9%), limited by their smaller sample sizes.^12,18^ The incidence of bacteremia in SCD has decreased over the past 30 years in conjunction with the introduction of prophylactic measures, disease-modifying medications, and vaccine development,^4,5,10,19^ but it still occurs and is higher than the risk of undetected bacteremia in febrile young children (age 3-36 months) without SCD who present to the ED.^20^ Our results focus on the risk of bacteremia among children with SCD and fever who have already arrived in a pediatric ED. The relatively low risk of bacteremia and the positive outcomes achieved even for children who were diagnosed with bacteremia who were initially discharged from the ED and later readmitted, may be useful for discussion among ED and acute care clinicians about the appropriate workup, treatment, and disposition of children with SCD and fever and whether a more targeted deployment of resources is feasible.

A better determination of risk factors for bacteremia would aid bedside risk stratification by the ED clinician. In our study we identified an increased risk of bacteremia in children and young adults with (1) a previous episode of bacteremia, (2) a previous episode of CLABSI, or (3) receiving apheresis. The latter 2 risk factors (CLABSI and apheresis) are specific to those who have indwelling CVCs and have been implicated as bacteremia risk factors in SCD previously.^12,21^ These risk factors might help ED clinicians decide how much workup to conduct, what treatment to provide, and whether to hospitalize a patient. Patients with SCD and a CVC require careful assessment and care in the ED, but CVC-related infections are iatrogenic, so these risk factors ought to spur attention and consideration from outpatient clinicians who care for and access indwelling CVCs in children with SCD.

The last major shift in care for febrile children with SCD occurred more than 30 years ago with the introduction of outpatient management if children were deemed low risk (ie, age >12 months, well appearing).^22^ In this cohort, 127 of 405 children with bacteremia were initially discharged and subsequently hospitalized with bacteremia. Only 1 of those children was admitted to the ICU, and none of them died. This supports the safety of this approach and suggests that further risk stratification may be possible and even a larger proportion of children with SCD and fever could have their care managed as outpatients after clinical and laboratory assessment in the ED. A large, prospectively derived and validated clinical decision rule for this population could provide the basis to change clinical practice.

In this large cohort, SCD genotype, age, race and ethnicity, sex, and insurance type were not associated with a greater risk of bacteremia in the ED. Prior prospective cohort studies have identified that children with hemoglobin SS (HbSS) disease and younger children have a greater risk of bacteremia.^4,5,23^ While these studies investigated bacteremia events in a whole single-center or multicenter cohort over time, our analysis was restricted to children arriving in the ED with fever. Other studies of febrile children with SCD did not find that HbSC was a risk factor for bacteremia,^12^ although one study found that age greater than 6 years was a risk factor.^21^ While children with HbSC may be at lower risk for bacteremia over the course of a lifetime, this fact is not useful for a clinician at bedside if the child has a similar risk for bacteremia as a child with HbSS when febrile in the ED.

Bacteremia is often associated with a concomitant infectious diagnosis,^5,23^ with some clinically apparent at the time of the initial ED evaluation. For instance, half of the cases of ACS identified in one clinical cohort were diagnosed after hospitalization for another reason.^24^ In this cohort, 21.0% of children and young adults with bacteremia had the concomitant diagnosis of ACS entered during their hospital encounter, but the rates of ACS in those without bacteremia with SCD presenting with fever was similar. Meningitis and osteomyelitis were rare diagnoses. Septic arthritis, osteomyelitis, and urinary tract infection had significantly higher rates of bacteremia, consistent with hematogenous seeding in osteomyelitis and septic arthritis or a urinary tract infection pyelonephritis pathway to the bloodstream. If symptoms of a more serious clinical syndrome are present during ED evaluation, ED clinicians should be aware of the risk of concurrent bacteremia or subsequent bacteremia.

### Limitations

This study is subject to several limitations as it is based on an administrative database. Namely, our data do not include clinician notes, physical examination findings, or laboratory test results. As we did not have access to blood culture results, we used *ICD-10* coding for the primary outcome of bacteremia, which may predispose to misclassification bias if, instead of bacteremia, these visits were given other codes, such as sepsis. It is also possible that coding for bacteremia may not have been performed in case of patient death (blood cultures may return as positive after death), and therefore we may have underestimated mortality. It is notable that 12.4% of patients included did not receive blood cultures, which we included in the denominator as some children and young adults transferred from an outside hospital likely had blood cultures performed before arrival at the PHIS institution, which we could not capture. This is evidenced by the fact that 33 of 405 (0.8%) children and young adults with a diagnosis of bacteremia did not have a blood culture obtained in the first 48 hours. Despite these limitations, the rate of bacteremia noted in this study is similar to that in smaller studies with blood culture results available. Because we did not have access to physical examination findings or vital signs, we used *ICD-10* coding for fever or the presence of a blood culture and antibiotic administration as a proxy for fever (standard of care for SCD fever workup), which is subject to the same limitations. We relied on *ICD-10* codes for genotype variable and cannot confirm the true genotype. However, SCD genotypes appeared to be consistent across encounters. Some of our risk factors were also subject to potential misclassification bias, in particular, CVC coding was based on the historical procedure code for placement of a CVC, which did not ensure that a CVC was present during the encounter. Our study was also limited to patients presenting to tertiary care pediatric hospitals and may not be generalizable to other settings; however, most children and young adults in the US with SCD are cared for in major metropolitan areas where children’s hospitals are usually found.^25^ Given the limitations of this study, current guidelines should be followed until a low-risk cohort can be further elucidated through cohort and prospective studies.

## Conclusions

In this cohort study of children and young adults with SCD presenting to the ED with fever or a workup consistent with fever, bacteremia was an uncommon diagnosis. A history of bacteremia, CLABSI, or apheresis appeared to increase the odds of bacteremia. Prospective studies on children and young adults with SCD presenting with fever are needed to develop decision models and risk stratification tools to refine our approach and avoid unnecessary antibiotic exposure and hospitalization in this population.

## Notes

Supplement 1. **eAppendix.** Case Definitions Based on *ICD-9*, *ICD-10*, and *CPT* Codes **eTable 1.** Risk Factors for Bacteremia in Multivariable Model Excluding No Blood Culture Obtained **eTable 2.** Sickle Cell Disease Cohort Characteristics and Outcomes Between Admitted vs Discharged Children at Index Visit

Supplement 2.
Data Sharing Statement
