# Supplementary material for: Risk of Bacteremia in Febrile Children and Young Adults With Sickle Cell Disease in a Multicenter Emergency Department Cohort
Source: JAMA Netw Open. 2023 Jun 20;6(6):e2318904. doi: 10.1001/jamanetworkopen.2023.18904 (PMC10282882; doi:10.1001/jamanetworkopen.2023.18904)
Supplement: Supplement 1. — eAppendix. Case Definitions Based on ICD-9, ICD-10, and CPT Codes eTable 1. Risk Factors for Bacteremia in Multivariable Model Excluding No Blood Culture Obtained eTable 2. Sickle Cell Disease Cohort Characteristics and Outcomes Between Admitted vs Discharged Children at Index Visit [file jamanetwopen-e2318904-s001.pdf]

Supplementary Online Content

Rineer S, Walsh PS, Smart LR, Harun N, Schnadower D, Lipshaw MJ. Risk of bacteremia in febrile children and young adults with sickle cell disease in a multicenter emergency department cohort. *JAMA Netw Open*. 2023;6(6):e2318904. doi:10.1001/jamanetworkopen.2023.18904

eAppendix. Case Definitions Based on *ICD-9*, *ICD-10*, and *CPT* Codes

eTable 1. Risk Factors for Bacteremia in Multivariable Model Excluding No Blood Culture Obtained

eTable 2. Sickle Cell Disease Cohort Characteristics and Outcomes Between Admitted vs Discharged Children at Index Visit

This supplementary material has been provided by the authors to give readers additional information about their work.

© 2023 Rineer S et al. *JAMA Network Open.*

<u>eAppendix. Case Definitions based on *ICD-9*, *ICD-10*, and *CPT* codes</u>

Sepsis: A039, A021, A207, A217, A227, A239, A241, A267, A280, A282, A327, A392, A393, A394, A40.x, A41.x, A427, B007, B377, P352, P36.x, P372, P375

Central line placement: CPT: 36555, 36556, 36557, 36568, 36569, 36570, 36571

Apheresis: CPT: 36511, 36512, 36513, 36514, 36515, 36516, 36517, 36518, 36519, 36520, 36521, 36522

Acute Chest Syndrome: D5701, D57211, D57411, D57431, D57451, D57811, J10x, J11x, J12x, J13x, J14x, J15x, J16x, J17x, J18x, 480x, 481x, 482x, 483x, 484x, 485x, 486x, 487x, 488x, 5173

CLABSI: T80211A, T80211D, T80211S

Bacteremia: R7881, 7907

Splenectomy: Z9081

Stroke: I63x, I693, 433x, 434x, 4389

Bone Marrow Transplant: Z9481

Fever: R5081, R50.9

Osteomyelitis: M86x

© 2023 Rineer S et al. *JAMA Network Open.*

eTable 1. Risk Factors for Bacteremia in Multivariable Model Excluding No Blood Culture Obtained[a]

	OR (95% CI)	P-value
Age (years)		
<1	0.95 (0.65-1.39)	0.83
1-5	0.98 (0.77-1.24)	0.97
>5	-	-
Female	1.06 (0.86-1.31)	0.56
Male	-	-
Genotype		
HbSC	0.75 (0.57-1.00)	0.53
HbSThalassemia[b]	0.78 (0.52-1.16)	0.77
Other/multiple	0.77 (0.38-1.57)	0.83
HbSS	-	-
Previous Diagnoses		
Bacteremia	1.69 (1.26-1.17)	<.01
Osteomyelitis	2.01 (1.17-3.46)	0.01
Splenectomy	0.79 (0.54-1.17)	0.24
Sepsis	1.13 (0.72-1.78)	0.59
CLABSI	6.65 (3.27-13.54)	<.01
CVC Placement	1.30 (0.86-1.99)	0.22
Apheresis	1.67 (1.16-2.41)	<.01

[a] Generalized linear mixed model accounting for repeat encounters and nested within hospitals

[b] HbS-Beta Zero or HbS-Beta Plus. CLABSI = Central Line Associated Blood Stream Infection, CVC = Central Venous Catheter

© 2023 Rineer S et al. *JAMA Network Open.*

eTable 2: Sickle Cell Disease Cohort Characteristics and Outcomes Between Admitted vs Discharged Children at Index Visit

Characteristic	Overall	Admitted	Discharged	P-value
N	405	276	129	
By age (%)				0.48
Less than 1 year		24 (8.7)	11 (8.5)	
1 to 5 years		84 (30.4)	47 (36.4)	
>5 years		168 (60.9)	71 (55.0)	
Male (%)		151 (54.7)	70 (54.3)	0.93
Genotype (%)				0.28
Hemoglobin SS		205 (74.3)	93 (72.1)	
Hemoglobin SC		42 (15.2)	24 (18.6)	
Hemoglobin S-Thalassemia[a]		25 (9.1)	8 (6.2)	
Other/Multiple		3 (1.1)	4 (3.1)	
Medical History and Patient Characteristics (%)				
Acute Chest Syndrome		157 (56.9)	75 (58.1)	0.81
Splenectomy		24 (8.7)	10 (7.8)	0.75
Bacteremia		47 (17.0)	23 (17.8)	0.84
Apheresis		34 (12.3)	11 (8.5)	0.26
Sepsis		20 (7.3)	4 (3.1)	0.12
Central Venous Catheter Placement		22 (8.0)	8 (6.2)	0.52
Stroke		13 (4.7)	5 (3.9)	0.80
Osteomyelitis		11 (4.0)	5 (3.9)	1.00
CLABSI		12 (4.4)	0	0.01

[a]HbS-Beta Zero or HbS-Beta Plus. ACS = Acute Chest Syndrome, CLABSI = Central Line Associated Blood Stream Infection, ICU = Intensive Care Unit, UTI = Urinary Tract Infection

© 2023 Rineer S et al. *JAMA Network Open.*
