# Supplementary material for: Risk of Bacteremia in Febrile Children and Young Adults With Sickle Cell Disease in a Multicenter Emergency Department Cohort
Source: JAMA Netw Open. 2023 Jun 20;6(6):e2318904. doi: 10.1001/jamanetworkopen.2023.18904 (PMC10282882; doi:10.1001/jamanetworkopen.2023.18904)
Supplement: Supplement 2. — Data Sharing Statement [file jamanetwopen-e2318904-s002.pdf]

Data Sharing Statement

Rineer. Risk of Bacteremia in Febrile Children and Young Adults With Sickle Cell Disease in a Multicenter Emergency Department Cohort. *JAMA Netw Open*. Published June 20, 2023. doi:10.1001/jamanetworkopen.2023.18904

Data
Data available: No
